# Liver transplant and impact on cerebral autoregulation: a systematic scoping review

**DOI:** 10.3389/frtra.2025.1753109

**Published:** 2026-01-19

**Authors:** Tommaso Rochat, Artida Ulaj, Frederic Sangla, Pia De Stefano, Herve Quintard

**Affiliations:** University Hospitals of Geneva, Geneva, Switzerland

**Keywords:** cerebral autoregulation, hemodynamic management, liver transplantation, orthotopic liver transplant, perioperative monitoring

## Abstract

**Background:**

Cerebral autoregulation (CA)—the brain's ability to maintain stable cerebral blood flow (CBF) despite changes in mean arterial pressure (MAP)—may be disrupted during orthotopic liver transplantation (OLT) due to profound metabolic, inflammatory, and hemodynamic alterations. Such impairment could increase the risk of cerebral hypoperfusion or hyperemia, yet its perioperative evolution and clinical implications remain unclear. This systematic scoping review aimed to synthesize current evidence on CA monitoring during OLT and to identify methodological gaps and potential clinical applications.

**Methods:**

A systematic scoping review was conducted according to Joanna Briggs Institute (JBI) guidelines. Studies including adult patients undergoing OLT with quantitative perioperative CA assessment were identified across PubMed, Embase, Scopus, Web of Science, and Medline.

**Results:**

Six studies (*n* = 99) met inclusion criteria. CA was assessed using diverse methods, including transcranial Doppler (TCD), near-infrared spectroscopy (NIRS), and derived indices such as the pressure reactivity index (PRx), mean flow index (Mx), cerebral oximetry index (COx), static cerebral autoregulation index (SCAI), and transfer function analysis (TFA). Monitoring phases and definitions of impaired CA varied widely. In acute liver failure, CA was commonly impaired pre-transplant and improved postoperatively, whereas findings in chronic liver disease were inconsistent. Only a few studies explored associations with neurological outcomes, yielding inconclusive results.

**Conclusions:**

CA appears to be dynamically affected during OLT, particularly in acute liver failure, but evidence remains limited by methodological heterogeneity and small sample sizes. Standardized, prospective studies are needed to clarify the role of CA monitoring and to determine whether individualized blood pressure management could improve neurological outcomes.

## Introduction

Cerebral autoregulation is a critical homeostatic mechanism that maintains stable cerebral blood flow (CBF) despite fluctuations in systemic blood pressure (1, 2). In patients with acute or chronic liver failure, especially those undergoing orthotopic liver transplantation, CA is frequently disrupted due to metabolic, inflammatory, and hemodynamic derangements (3–6). When autoregulation is impaired, cerebral perfusion becomes pressure-dependent, potentially predisposing the brain to ischemic or hyperemic injury during periods of hypotension or hypertension. After traumatic brain injury, impaired CA has been associated with poorer neurological outcomes, reinforcing its importance as a prognostic and therapeutic target (7, 8).

Despite growing interest, evidence on CA before, during, and after liver transplantation remains inconsistent. Some studies show impaired CA preoperatively with postoperative recovery, whereas others report preserved autoregulation throughout the surgical phases (3, 5, 9–12). Such discrepancies likely reflect variations in monitoring techniques, interpretation criteria, and perioperative conditions.

Multiple methodological approaches contribute further variability. Static measures, such as the Static Cerebral Autoregulation Index (SCAI), evaluate cerebrovascular responses to induced MAP changes, with values >0.6 indicating preserved autoregulation (13). Dynamic indices—including the Pressure Reactivity Index (PRx) and Mean Flow Index (Mx) (14), quantify correlations between spontaneous MAP fluctuations and intracranial pressure or cerebral blood flow velocity; values <0.3 indicate intact, and ≥0.3–0.4 impaired, autoregulation. The Cerebral Oximetry Index (COx), derived from near-infrared spectroscopy, incorporates both flow and metabolic information (15, 16), while Transfer Function Analysis (TFA) assesses gain and phase relationships between MAP and CBFV across frequency bands (2, 17–19). Limited assessments of pre- and postoperative periods also contribute to heterogeneity.

This scoping review synthesizes current evidence on CA in OLT, examining perioperative changes, methodological differences, and associations with neurological outcomes. By integrating physiological and technical perspectives, the review aims to clarify the potential role of CA monitoring in improving risk stratification and guiding individualized blood pressure management during liver transplantation.

## Methods

This systematic scoping review was conducted in accordance with the Joanna Briggs Institute (JBI) methodology for scoping reviews (20) PRISMA-ScR reporting items, aiming to map the evidence on CA in the setting of liver transplantation.

### Participants

Studies were considered eligible if they included adult patients (≥18 years) undergoing orthotopic liver transplantation, irrespective of the underlying liver disease etiology (acute liver failure or chronic end-stage liver disease). Both sedated and non-sedated patients in intensive care or intraoperative settings were included.

### Context

This review focused on the perioperative period, encompassing preoperative (before anesthesia induction), intraoperative, and post operative phases (after transplantation), during which CA was assessed. The review specifically examined methods used to quantify cerebral autoregulation and its changes during all the periods.

### Type of sources

Eligible sources included peer-reviewed original studies, including observational cohort studies, prospective pilot studies, and retrospective analyses. Case reports, editorials, reviews, and preclinical studies were excluded. Grey literature and conference abstract were not screened.

### Search strategy

An initial search was conducted in PubMed, Embase, Web of Science, Scopus, and Medline from February 2025 to 01. April 2025, with the goal of identifying studies evaluating CA during liver transplantation. Only studies published in English and French were included. Backward and forward citation tracking were performed to identify additional relevant references not retrieved by the initial database search.

**Figure A1:**
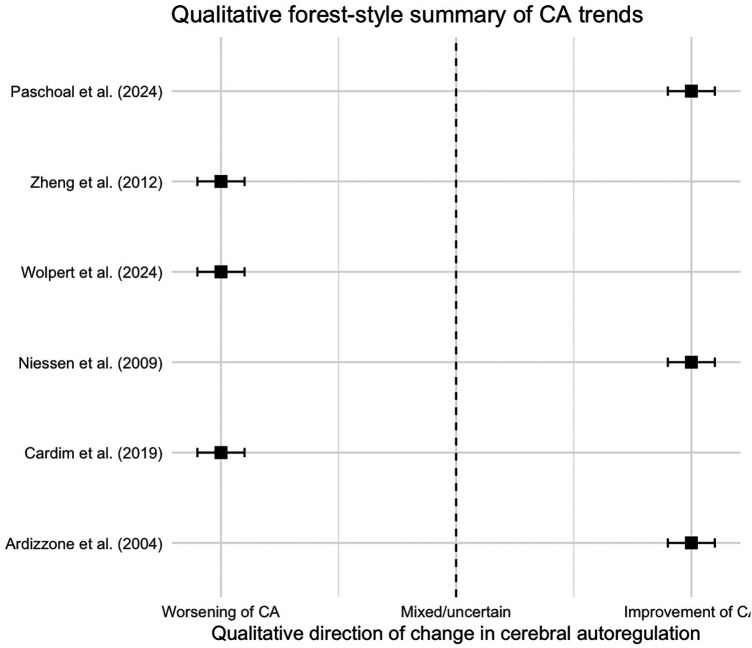
Qualitative Forest style summary of perioperative cerebral autoregulation trends reported in the included studies, illustrating the direction of change during OLT.

Studies were required to report quantitative assessment of cerebral autoregulation and must include monitoring during at least one perioperative phase. Studies without CA assessment or without clearly defined timing of monitoring were excluded. A detailed search strategy for each database is presented in Appendix Figure A1.

### Studies

**Figure 1:**
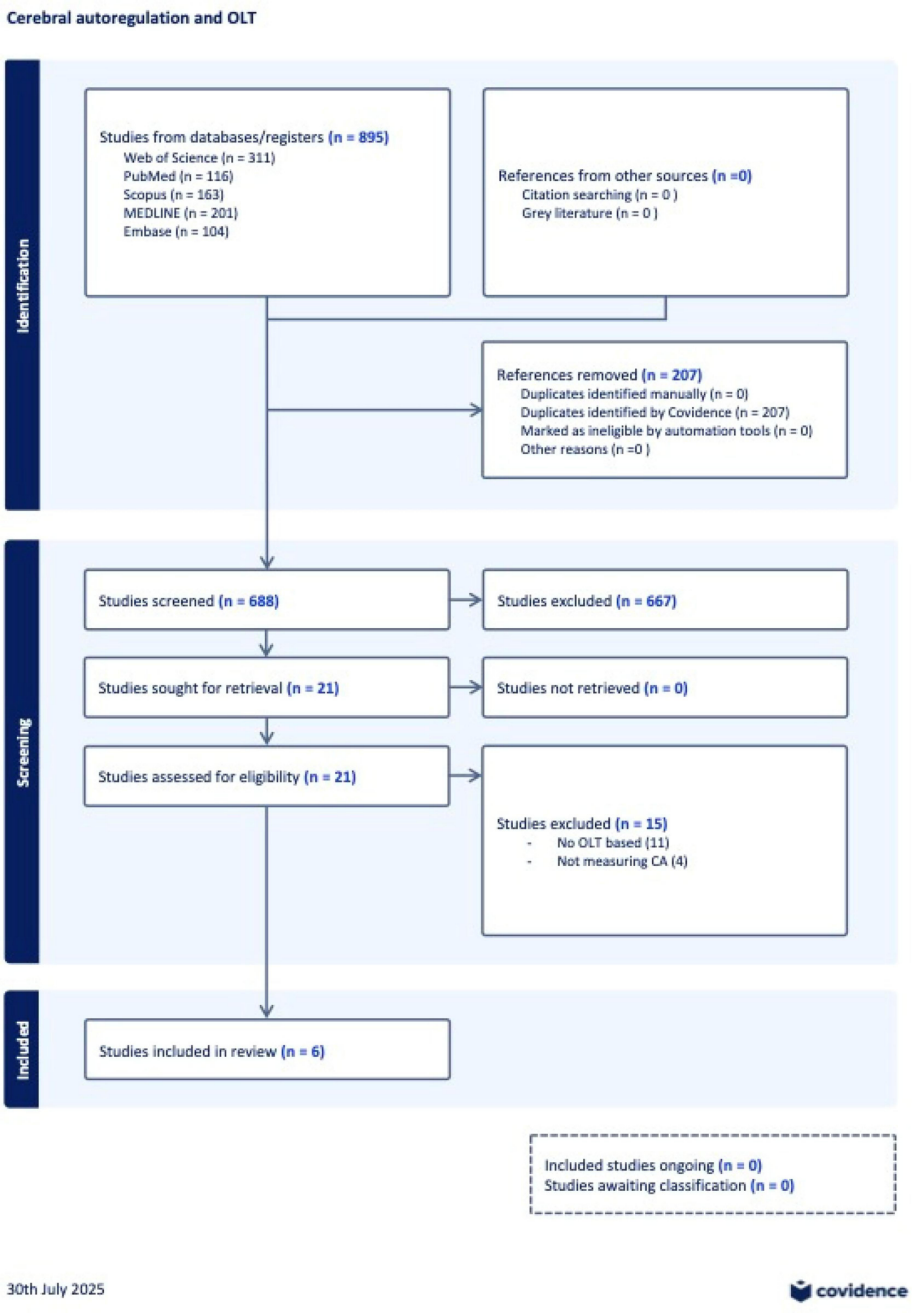
PRISMA-ScR study selection process for evidence on cerebral autoregulation in orthotopic liver transplantation. Adapted from Page et al. (doi: 10.1136/bmj.n71) using Covidence systematic review software, Veritas Health Innovation, Melbourne, Australia, available at www.covidence.org.

Following deduplication (Covidence software), all titles and abstracts were screened independently by two reviewers. Full texts of potentially relevant articles were assessed for eligibility. Discrepancies were resolved through discussion or by a third reviewer when necessary. A total of six studies met the inclusion criteria (Figure 1).

### Data extraction

Data were extracted by two independent reviewers (T.R. and A.U.) using the Covidence data extraction tool (Covidence systematic review software, Veritas Health Innovation, Melbourne, Australia) developed *a priori* and refined following preliminary screening to ensure clarity. Extracted data included participant characteristics, cerebral autoregulation assessment methods, timing of monitoring (pre-, intra-, postoperative), autoregulation indices used, anesthetic methods and outcomes. Any disagreements in data extraction were resolved through discussion or arbitration by a third reviewer.

### Risk of bias assessment

Risk of bias was evaluated for each eligible study. For observational studies, the modified Newcastle–Ottawa scale tool was used (21).

Appendix Table A1 resumes the methodological quality scores of each study with the six observational studies obtaining a NOS above the quality threshold set at three stars on nine.

## Results

This review includes six studies (9–11, 22–24): four prospective observational studies and two retrospective analyses. The studies were conducted between 2004 and 2024 in Brazil, the United States, Germany, Denmark, and the United Kingdom. Across the studies, a total of 99 adult patients undergoing orthotopic liver transplantation were included. Sample sizes ranged from 6 to 33 patients. Patient populations encompassed both fulminant hepatic failure (FHF) and chronic liver disease.

Cerebral autoregulation (CA) was assessed during different perioperative phases: three studies focused exclusively on intraoperative monitoring, while two extended measurements to the postoperative period, and only one (Paschoal et al.) provided both pre- and multi-day postoperative data. A wide variety of monitoring techniques and autoregulatory indices were used, including TCD,NIRS, non-invasive intracranial pressure (nICP) estimation, and derived indices such as PRx, Mx, COx, SCAI, and transfer function analysis. Only two studies applied continuous high-resolution waveform analysis. The definitions and thresholds for impaired autoregulation also varied substantially (e.g., Mx ≥ 0.4, SCAI <0.6), and only three studies attempted to correlate autoregulation impairment with clinical or neurological outcomes. A detailed overview of each study is provided below and summarized in Table 1.

**Table 1:** Summary of six studies (*n* = 99) assessing cerebral autoregulation (CA) during orthotopic liver transplantation.

Study	Country	Design	N° of patients	Sex (M/F)	Age (Mean or Median)	MELD Score (Mean or Median)	Hepatic Encephalopathy	Liver failure type	Etiology of liver failure (n°)	CA monitoring method	Perioperative period	Phases assessed	Cerebral parametres	Anesthesia	CO_2_ control	Outcome
Ardizzone et al. (9)	Italy/USA	Prospective observational	6	5 M	Mean 33.8y (SD 13.1)	N/A	Grade III–IV (n° or %not specified)	Acute	HBV(2); Intoxication (4)	TCD	Intra-operative	Paleohepatic, Neohepatic	CBFV, a-jDO_2_, eCMRO_2_	Propofol + fentanyl (TIVA)	EtCO_2_ maintained at ∼35 mmHg	N/A
Cardim et al. (10)	UK	Retrospective observetional	6	4 M	Median 56y (range 51–65)	N/A	N/A	Acute and chronic	PSC(2); Ar(2);HCV(1);Crypto(1)	TCD	Intra-operative	Dissection, Anhepatic, Reperfusion	nICP, nCPP, PI, CrCP, DCM, Mxa, nPRx	Remifentanil + atracurium + isoflurane	EtCO_2_ monitored (range not specified)	No correlation
Nissen et al. (23)	Denmark	Prospective observational	33	19 F	Median 49y (range 19–63)	N/A	N/A	Acute and chronic	Ar(6);PBC(5);PSC(4);ReT(4); HCV(4);ALF(2);HCC(2);Crypto(6)	NIRS (ScO_2_)	Intra-operative	No phase division	ScO_2_ vs. MAP correlation	Fentanyl + propofol + remifentanil + cisatracurium	EtCO_2_ at 26–34 mmHg	N/A
Paschoal et al. (11)	Brazil	Prospective observational	25	19 F	Mean 33y (SD 13.1)	N/A	Grade I(8%), II(8%), III(56%), IV(28%)	Acute	Unknown(19);Wilson(2);Intox(4)	TCD	Pre/Post operative	Pre- and POD 1–3	SCAI	Propofol + fentanyl	Not mentioned	Not formally analyzed
Wolpert et al. (24)	Germany	Prospective observational	20	13 M	Mean 52y (SD 13)	25.5 (17–30)	N/A	Acute and chronic	N/A	TCD	Intra-operative	Dissection, Anhepatic, Reperfusion	TFA (gain and phase)	Sufentanil + propofol + sevoflurane	paCO_2_ at 35–45 mmHg	No correlation
Zheng et al. (22)	USA	Prospective observational	9	2 F	Mean 51y (SD 14)	17.9 ± 3	N/A	Chronic	Ar(1);HCC(4);HCV(1);Other(1);Autoim(1);reT(1)	TCD + NIRS	Intra-operative	Dissection, Anhepatic, Reperfusion	Mx, COx	Propofol + fentanyl + vecuronium + isoflurane	EtCO_2_ at 35–45 mmHg	Yes

CA, cerebral autoregulation; OLT, orthotopic liver transplantation; TCD, transcranial Doppler; NIRS, near-infrared spectroscopy; SCAI, static cerebral autoregulation index; PRx, pressure reactivity index; Mx, mean flow index; COx, cerebral oximetry index; nPRx, non-invasive PRx; POD, postoperative day. PI, pulsatility Index; CrCP, Critical closing Pressure; Mxa, Mean Flow Index; MAP, Mean arterial Pressure; CBFV, cerebral flow velocity; DCM, Diastolic Closing Margin; nCPP, Non-invasive Cerebral Perfusion Pressure; Ar, alcohol related; ReT, Re-Transplant; HCC, Hepatocellular Carcinoma; PSC, Primary Sclerosing Cholangitis; PBS, Primary Biliary Cholangitis.

Appendix Table A2 and Figure A1 provides a qualitative summary of the perioperative trends in cerebral autoregulation reported across the included studies, highlighting whether each investigation described improvement, preservation, or impairment of CA, while acknowledging that direct comparisons remain limited by mixed patient populations and heterogeneous assessment methodologies.

Below we present the salient feature of the studies:

Paschoal et al. conducted a prospective study in 25 patients with fulminant hepatic failure (FHF) undergoing OLT, evaluating cerebral autoregulation using the static cerebral autoregulation index (SCAI). The mean age was 33.8 years, and 76% were female.

Anesthesia was administered with propofol and fentanyl, and all patients were mechanically ventilated. Recorded physiological parameters included mean arterial pressure, end-tidal CO_2_ (EtCO_2_), and hemoglobin concentration. Cerebral blood flow velocity (CBFv) in the middle cerebral arteries was measured using transcranial Doppler (TCD). SCAI was calculated from the change in cerebrovascular resistance (CVR) in response to a norepinephrine-induced MAP increase (20–30 mmHg). Values > 0.6 indicated preserved autoregulation.

SCAI was assessed pre-transplant and on postoperative days (POD) 1, 2, and 3. A significant improvement in SCAI was observed after transplantation (*p* = 0.005), with peak values on POD 3 (*p* = 0.006). Pre-transplant, only one patient had SCAI > 0.6; by POD 2, 41.7% exceeded this threshold. EtCO_2_ and hemoglobin levels showed no significant changes over time.

In the article by Zheng et al. nine patients were enrolled in a prospective feasibility study using simultaneous TCD and NIRS during all phases of OLT. CA was quantified using Mx and COx (Pearson's correlation between MAP and CBF velocity or cerebral oxygenation, respectively). Impaired CA (Mx ≥ 0.4) was observed in one patient throughout and intermittently in three others. Mx and COx were strongly correlated (*p* = 0.0029), even in patients with elevated bilirubin. Impaired CA was significantly associated with MELD > 15 (*p* = 0.015) and postoperative neurologic complications (*p* < 0.0001).

Wolpert et al. conducted a prospective observational pilot study in 20 patients assessing dynamic cerebral autoregulation (dCA) using transcranial Doppler-based transfer function analysis. The cohort included patients with acute and chronic liver failure.

Anesthesia was conducted with sufentanil, propofol, rocuronium, and sevoflurane, with ventilation adjusted to maintain paCO_2_ between 35 and 45 mmHg. Recorded physiological variables included MAP, cardiac index, heart rate, paCO_2_, and norepinephrine dosage.

TCD was used to measure cerebral blood velocity (CBv) in the middle cerebral artery, synchronized with invasive arterial blood pressure. Dynamic CA was evaluated using frequency-domain transfer function analysis, calculating gain and phase shift across very low (0.02–0.07 Hz), low (0.07–0.2 Hz), and high (0.2–0.5 Hz) frequency bands. Lower gain and higher phase indicate more intact autoregulation.

In the very low-frequency band, gain significantly increased from the preparation phase (mean 0.18 ± 0.27) to both the anhepatic (1.01 ± 1.15; *p* = 0.004) and reperfusion phases (0.80 ± 0.93; *p* = 0.006). In the low-frequency band, gain rose from 0.19 ± 0.25 to 1.22 ± 1.96 during reperfusion (*p* = 0.046). Phase values did not differ significantly across phases in any frequency band.

No associations were found between dCA metrics and MELD score, hepatic encephalopathy, or postoperative outcomes. These results highlight phase-dependent dCA impairment, particularly during the anhepatic and reperfusion periods.

The work of Niessen et al. investigated cerebral autoregulation in a cohort of 33 patients using NIRS to monitor frontal lobe cerebral oxygenation (ScO_2_). The study population included patients with acute and chronic liver failure.

Anesthesia was administered using propofol and fentanyl for a median duration of 6 h and 40 min. To assess cerebral autoregulation, ScO_2_ values were analyzed in relation to MAP throughout the procedure. For each patient, a computer-assisted curve-fitting algorithm was applied to the full range of recorded MAP values. If ScO_2_ remained stable across the MAP range, autoregulation was considered preserved.

Of the 31 patients with analyzable NIRS data (two excluded due to interference from hyperbilirubinemia), three demonstrated a continuous positive relationship between ScO_2_ and MAP, consistent with impaired autoregulation. In 20 patients, ScO_2_ remained stable at MAP values as low as 42 mmHg (median 55 mmHg, range 42–66), indicating preserved autoregulation within those ranges. In the remaining eight patients, a distinct lower limit of autoregulation was identified, with a median threshold MAP of 69 mmHg (range 50–90). Correlation coefficients (r) for these patients ranged from 0.351 to 0.889, all statistically significant (*p*-values 0.024 to <0.001).

A retrospective analysis conducted at Addenbrooke's Hospital evaluated six patients with acute or chronic liver failure undergoing orthotopic liver transplantation (Cardim et al.). General anesthesia included intravenous remifentanil and atracurium with low-dose isoflurane. Continuous intraoperative monitoring comprised invasive arterial pressure, EtCO_2_, and bilateral TCD of middle cerebral artery flow velocity (FV).

A multimodal neuromonitoring approach was applied, using synchronized TCD and arterial pressure signals to derive non-invasive intracranial pressure (nICP), cerebral perfusion pressure (nCPP), pulsatility index (PI), critical closing pressure (CrCP), diastolic closing margin (DCM), Mxa, and non-invasive pressure reactivity index (nPRx). Measurements were evaluated across dissection (T0), anhepatic (T1), and reperfusion (T2) phases.

nCPP and DCM significantly decreased over time (*p* = 0.03), while PI remained elevated and CrCP increased but stayed below ABP. Mxa values were elevated across all phases, increasing from 0.56 at T0 to 0.72 at T2, indicating impaired and progressively worsening autoregulation. Median Mxa values were 0.56 (IQR 0.51–0.62) at T0, 0.67 (IQR 0.56–0.70) at T1, and 0.72 (IQR 0.55–0.81) at T2, reflecting a progressive increase from dissection to reperfusion.

Finally, Ardizzone et al. conducted a prospective study evaluating cerebral autoregulation and metabolism in six comatose patients with fulminant hepatic failure. All patients received anesthesia with propofol and fentanyl. EtCO_2_ as well as MAP, arterial oxygen saturation, esophageal temperature, arterial pH, and hemoglobin levels were continuously monitored.

Cerebral autoregulation was assessed using transcranial Doppler ultrasound to measure middle cerebral artery blood flow velocity (CBFV), and a slow phenylephrine infusion was used to incrementally increase MAP. Cerebral metabolism was estimated by calculating the arterial–jugular venous oxygen content difference (a-jDO_2_).

Cerebral hemodynamic and metabolic changes were analyzed during two defined intraoperative periods: the paleohepatic phase (first hour of surgery) and the neohepatic phase (last hour, post-reperfusion). During the paleohepatic phase, all six patients exhibited impaired autoregulation (r range: 0.71–0.90) and reduced cerebral oxygen extraction with a-jDO_2_ < 4 Vol%. In the neohepatic phase, autoregulation improved in all patients (r range: 0.10–0.48), and a-jDO_2_ increased to normal in four patients. The estimated cerebral metabolic rate for oxygen (eCMRO_2_) rose in all patients, with relative increases ranging from +12.5% to +114%.

There was no statistically significant difference between the two phases in systemic variables including EtCO_2_, MAP, temperature, pH, and hemoglobin.

## Discussion

The data analyzed and presented in the articles of this systematic scoping review indicate that the effect of OLT on cerebral autoregulation remains controversial.

### Acute vs. chronic liver failure

The heterogeneity in reported findings on cerebral autoregulation during OLT likely reflects the distinct pathophysiologic mechanisms underlying acute and chronic liver failure.

In acute liver failure, CA impairment arises from a reversible cascade in which hyperammonemia, systemic inflammation, and accumulation of neurotoxic metabolites released during massive hepatocyte necrosis trigger astrocytic swelling, cytotoxic edema, and increased intracranial pressure. These processes transiently disrupt cerebrovascular pressure reactivity, leading to a pressure-passive cerebral circulation. Following successful transplantation and restoration of graft function, normalization of ammonia levels and cerebral metabolism typically results in partial or complete recovery of autoregulatory function (Paschoal et al., 2024; Ardizzone et al., 2004) (6, 25).

Conversely, chronic liver failure produces more persistent cerebrovascular alterations. Long-term endothelial dysfunction, portal hypertension, systemic vasodilation, and excess nitric-oxide (NO) bioavailability induce NO-mediated vasoplegia that chronically attenuates vascular tone and responsiveness. This sustained vasodilatory state effectively flattens the autoregulatory curve, reducing the brain's capacity to buffer blood-pressure fluctuations and contributing to the wide interindividual variability observed across studies (12, 26–28).

In this context, heterogeneity among reported CA findings appears mechanistically grounded. ALF reflects *a* reversible disturbance of pressure reactivity, whereas chronic disease represents a structural and biochemical remodeling of the cerebrovascular bed. The relationship between hepatic encephalopathy and CA remains uncertain; Wolpert et al. found no association, whereas Zheng et al. linked impaired CA with neurological complications without correlation to HE severity. Whether HE directly influences CA in OLT patients therefore remains unresolved.

Finally, none of the included studies assessed optic nerve sheath diameter (ONSD), despite its potential relevance in acute liver failure where the risk of cerebral edema and elevated intracranial pressure is highest; future research is needed to clarify how impaired autoregulation interacts with rising ICP in this setting and whether multimodal monitoring, including ONSD, could enhance perioperative neuroprotection.

### Anesthesia and liver transplantation phases

Variability in anesthetic management and the segmentation of intraoperative phases may had an impact on reported cerebral autoregulation findings across studies.

While most investigations employed total intravenous anesthesia (TIVA) using propofol and fentanyl two studies (Cardim et al. and Wolpert et al.) utilized sevoflurane. As a volatile anesthetic with known dose-dependent cerebral vasodilatory properties, sevoflurane may impair CA by reducing cerebrovascular resistance and altering the pressure–flow relationship. This pharmacologic effect warrants consideration when interpreting results from studies incorporating volatile agents, as it may confound the true state of cerebrovascular regulation.

Ventilatory strategies also differed across studies. In some, such as those by Niessen, Zheng, and Cardim, EtCO_2_ was closely monitored and maintained within a normocapnic range (35–45 mmHg). However, several reports did not specify CO_2_ management protocols. Given the potent vasodilatory influence of carbon dioxide on cerebral vessels, inconsistencies in EtCO_2_ control represent a significant methodological confounder in the assessment of CA.

Moreover, not all studies evaluated CA dynamics in relation to the distinct intraoperative phases of orthotopic liver transplantation. Only four out of six segmented measurements across the dissection, anhepatic, and reperfusion periods. These phases are characterized by marked hemodynamic and metabolic transitions that likely influence cerebral perfusion. Studies that incorporated phase-specific analyses demonstrated clear temporal variation in autoregulatory indices, highlighting the importance of structured, time-resolved monitoring protocols for the accurate characterization of CA during liver transplantation.

Finally, emerging machine perfusion technologies may alter reperfusion physiology and consequently influence perioperative CA measurements.

### Cerebral autoregulation measurement

The techniques and indices used to evaluate CA varied substantially across studies, contributing to discrepancies in findings and interpretation.

Each method captures a distinct physiological aspect of CA. Static indices, such as the Static Cerebral Autoregulation Index (SCAI*)* used by Paschoal et al., assess the vascular response to controlled, step changes in mean arterial pressure, representing static autoregulation. In contrast, dynamic indices, including PRx and *Mx* (applied by Zheng et al. and Cardim et al.), quantify the correlation between spontaneous slow fluctuations in MAP and either intracranial pressure (PRx) or cerebral blood flow velocity (Mx), reflecting dynamic autoregulatory capacity. The COx index, derived from near-infrared spectroscopy, similarly correlates MAP with cerebral oxygen saturation (*Δ*ScO₂). Because ScO₂ reflects both cerebral blood flow and oxygen metabolism, COx is considered a mixed dynamic index, capturing the combined influence of flow and metabolic reactivity on cerebral oxygenation. Transfer function analysis (TFA*)*, as used by Wolpert et al., provides a frequency-domain approach, estimating gain and phase shift between MAP and cerebral flow oscillations across defined frequency bands.

Reported thresholds for impaired autoregulation varied accordingly—typically SCAI < 0.6, Mx or PRx ≥ 0.3–0.4, and COx ≥ 0.3—complicating cross-study comparison.

Only a few studies tracked temporal evolution of CA: Paschoal et al. observed progressive postoperative recovery, whereas Zheng et al. and Wolpert et al. described intraoperative phase-specific variations.

Notably, Niessen et al. and Zheng et al. attempted to define the lower limit of autoregulation (LLA)—the MAP threshold below which cerebral blood flow becomes pressure-dependent—which varied widely among patients (50–90 mmHg), underlining the importance of individualized hemodynamic targets during liver transplantation (29).

### Outcome associations

Only a limited number of studies examined whether CA impairment was associated with neurological or clinical outcomes.

Zheng et al. reported that patients with impaired autoregulation (Mx or COx ≥ 0.4) experienced postoperative neurological events, including seizures and stroke, and that these impairments were associated with higher MELD scores. In contrast, Wolpert and Cardim did not find significant associations between CA indices and outcome measures such as ICU stay, hospital stay, or mortality. While Paschoal et al. observed clinical improvement alongside SCAI recovery, outcome data were not formally analyzed.

Overall, the prognostic significance of impaired CA in the OLT setting remains underexplored. Additional studies with standardized outcomes and larger cohorts are needed to determine whether perioperative CA monitoring can serve as a predictive tool for neurological complications and guide individualized blood pressure management.

## Limitations

This review is limited by the small number of eligible studies and their heterogeneous designs, which preclude robust generalization. The included cohorts varied widely in liver failure etiology (acute vs. chronic), clinical context, and perioperative monitoring windows. Methodological inconsistencies, including diverse autoregulatory indices, definitions of impairment and monitoring modalities, further hinder direct comparisons and limit synthesis. Moreover, most studies had small sample sizes and lacked longitudinal follow-up, particularly in the postoperative period, restricting insight into the temporal dynamics of cerebral autoregulation.

## Conclusions

Cerebral autoregulation appears to be affected and dynamically modulated during orthotopic liver transplantation, particularly in patients with acute liver failure. Nevertheless, substantial heterogeneity in study methodologies, monitoring techniques, and patient populations limits cross-study comparability. Beyond its descriptive value, CA monitoring may hold clinical relevance. Future studies should explore whether individualized control of MAP—especially in patients with hepatic encephalopathy or impaired autoregulation—could help reduce neurological complications. Multimodal, noninvasive approaches combining transcranial Doppler and near-infrared spectroscopy may offer a practical alternative to invasive monitoring. Moreover, integrating ONSD with CA indices such as TCD-derived Mx or NIRS-derived COx could enable comprehensive multimodality monitoring both in the operating room and in the immediate postoperative period, offering valuable insight into the interaction between autoregulatory failure and evolving intracranial hypertension. Finally, the potential influence of volatile anesthetics on cerebrovascular reactivity and autoregulatory function warrants further investigation. Standardized, prospective studies are needed to clarify whether CA-guided hemodynamic management can improve neurological outcomes in OLT.

## Appendix A

PUBMED:

(“liver transplantation"[MeSH Terms] OR (“liver"[All Fields] AND “transplantation"[All Fields]) OR “liver transplantation"[All Fields]) AND ((“cerebrally"[All Fields] OR “cerebrum"[MeSH Terms] OR “cerebrum"[All Fields] OR “cerebral"[All Fields] OR “brain"[MeSH Terms] OR “brain"[All Fields]) AND (“autoregulate"[All Fields] OR “autoregulated"[All Fields] OR “autoregulates"[All Fields] OR “autoregulating"[All Fields] OR “autoregulations"[All Fields] OR “autoregulative"[All Fields] OR “autoregulator"[All Fields] OR “autoregulators"[All Fields] OR “homeostasis"[MeSH Terms] OR “homeostasis"[All Fields] OR “autoregulation"[All Fields]))

MEDLINE:

(exp “liver transplantation"/ OR (liver.af. AND transplantation.af.) OR “liver transplantation”.af.) AND ((cerebrally.af. OR exp cerebrum/OR cerebrum.af. OR cerebral.af. OR exp brain/ OR brain.af.) AND (autoregulate.af. OR autoregulated.af. OR autoregulates.af. OR autoregulating.af. OR autoregulations.af. OR autoregulative.af. OR autoregulator.af. OR autoregulators.af. OR exp homeostasis/ OR homeostasis.af. OR autoregulation.af.))

EMBASE:

(‘liver transplantation'/exp OR (liver AND transplantation) OR ‘liver transplantation’) AND ((cerebrally OR cerebrum/exp OR cerebrum OR cerebral OR brain/exp OR brain) AND (autoregulate OR autoregulated OR autoregulates OR autoregulating OR autoregulations OR autoregulative OR autoregulator OR autoregulators OR homeostasis/exp OR homeostasis OR autoregulation))

Web of Science:

(ALL="liver transplantation” OR (ALL = liver AND ALL = transplantation) OR ALL="liver transplantation”) AND ((ALL = cerebrally OR ALL = cerebrum OR ALL = cerebrum OR ALL = cerebral OR ALL = brain OR ALL = brain) AND (ALL = autoregulate OR ALL = autoregulated OR ALL = autoregulates OR ALL = autoregulating OR ALL = autoregulations OR ALL = autoregulative OR ALL = autoregulator OR ALL = autoregulators OR ALL = homeostasis OR ALL = homeostasis OR ALL = autoregulation))

SCOPUS:

(INDEXTERMS(”liver transplantation”) OR (ALL(liver) AND ALL(transplantation)) OR ALL(”liver transplantation”)) AND ((ALL(cerebrally) OR INDEXTERMS(cerebrum) OR ALL(cerebrum) OR ALL(cerebral) OR INDEXTERMS(brain) OR ALL(brain)) AND (ALL(autoregulate) OR ALL(autoregulated) OR ALL(autoregulates) OR ALL(autoregulating) OR ALL(autoregulations) OR ALL(autoregulative) OR ALL(autoregulator) OR ALL(autoregulators) OR INDEXTERMS(homeostasis) OR ALL(homeostasis) OR ALL(autoregulation)))

**Table A1:** Newcastle Ottawa Scale (NOS)for risk of bias assessment for the included studies in this review.

Study	Study design	Selection				Comparability of cohorts		Outcome			NOS
		Representat-iveness of sample	Non exposed cohort	Ascertain-ement	Outcome not present at start			Assessment	Time of follow-up	Adequacy of follow-up	
						Main factor	Addition-nal factor				
Ardizzone et al. (9)	Prospective	*	/	*	*	*	/	*	/	*	6/9
Cardim et al. (10)	Retrospective	*	/	*	*	*	/	*	/	*	6/9
Nissen et al. (23)	Prospective	*	/	*	*	*	/	*	*	*	7/9
Paschoal et al. (11)	Prospective	*	/	*	*	*	/	*	*	*	7/9
Wolpert et al. (24)	Prospective	*	/	*	*	*	/	*	*	*	7/9
Zheng et al. (22)	Prospective	*	/	*	*	*	/	*	/	*	6/9

Risk of bias using NOS evaluates risk of bias in the six observational studies.

**Table A2:** Summary of perioperative cerebral autoregulation (CA) trends reported across included studies.

Study	Liver Failure Type	Perioperative period	General Trend of CA
Paschoal et al. (11)	Acute	Pre/Post operative	Improved post-transplant
Zheng et al. (22)	Acute and chronic	Intra-operative	Mostly impaired pre and during trasnplant
Wolpert et al. (24)	Acute and chronic	Intra-operative	Worsened during anhepatic/reperfusion phases
Nissen et al. (23)	Acute	Intra-operative	Mostly preserved
Cardim et al. (10)	Acute and chronic	Intra-operative	Progressively impaired intraoperatively
Ardizzone et al. (9)	Chronic	Intra-operative	Improved post-reperfusion

Each study is categorized by liver failure type, perioperative monitoring period, and the overall direction of CA changes. Although these trends offer a general comparative overview, interpretation remains limited by heterogeneous patient populations and the use of different CA assessment methods across studies.
